# Dynamic Power-Saving Method for Wi-Fi Direct Based IoT Networks Considering Variable-Bit-Rate Video Traffic

**DOI:** 10.3390/s16101680

**Published:** 2016-10-12

**Authors:** Meihua Jin, Ji-Young Jung, Jung-Ryun Lee

**Affiliations:** School of the Electrical Engineering, Chung-Ang University, 84 Heukseok-ro, Dongjak-gu, Seoul 06974, Korea; mhkim0705@cau.ac.kr (M.J); jiyoung@cau.ac.kr (J.-Y.J.)

**Keywords:** Internet of Things (IoT), Wi-Fi Direct, power-saving, video traffic

## Abstract

With the arrival of the era of Internet of Things (IoT), Wi-Fi Direct is becoming an emerging wireless technology that allows one to communicate through a direct connection between the mobile devices anytime, anywhere. In Wi-Fi Direct-based IoT networks, all devices are categorized by group of owner (GO) and client. Since portability is emphasized in Wi-Fi Direct devices, it is essential to control the energy consumption of a device very efficiently. In order to avoid unnecessary power consumed by GO, Wi-Fi Direct standard defines two power-saving methods: Opportunistic and Notice of Absence (NoA) power-saving methods. In this paper, we suggest an algorithm to enhance the energy efficiency of Wi-Fi Direct power-saving, considering the characteristics of multimedia video traffic. Proposed algorithm utilizes the statistical distribution for the size of video frames and adjusts the lengths of awake intervals in a beacon interval dynamically. In addition, considering the inter-dependency among video frames, the proposed algorithm ensures that a video frame having high priority is transmitted with higher probability than other frames having low priority. Simulation results show that the proposed method outperforms the traditional NoA method in terms of average delay and energy efficiency.

## 1. Introduction

With the arrival of the era of Internet of Things (IoT), the widespread use of internet-enabled devices has led to a drastic increase in mobile data traffic. In recent years, there has been research on communication algorithms considering various data traffic characteristics for IoT networks [1,2,3,4,5,6]. Qinghe et al. applied the ultra-narrow band (UNB) technique to wireless clinical networks and proposed the corresponding massive access scheme, which is called hybrid periodic-random massive access (HPRMA), for co-existing periodic and random services [1]. In HPRMA, the number of UNB channels allocated to periodic and random services can be dynamically tuned according to traffic load information and diverse services can be simultaneously aligned with their periodic requirements. Hongliang et al. proposed a traffic-aware access class barring (TAACB) scheme to improve the scalability of machine-to-machine networks [2]. In TAACB, it is possible to accommodate more devices ensuring low access delay by regulating the parameter of access probability based on network traffic load. Qinghe et al. proposed the peer-to-peer share enabled routing schemes over multi-hop interference-constrained device-to-device networks, where multiple subscribers attempt to download the common data load from multiple distributed servers [3]. Maria et al. proposed a novel traffic aware scheduling algorithm (TASA) by extending the theoretically well-established graph theory methods based on network topology and traffic load [4]. Navrati et al. identified the complexity of the optimal traffic awareness in cloud radio access networks and designed a framework for traffic-aware energy optimization [5]. Nicola et al. presented a new decentralized traffic-aware scheduling algorithm, which is able to construct optimal multi-hop schedules in a distributed fashion, and proved its effectiveness by means of simulation results in IoT-compliant multi-hop networks [6]. In particular, to prevent base stations from being overloaded owing to the rapid increase in data traffic, there has been research on traffic load balancing methods using direct communication between mobile devices [7]. In this context, it is believed that Wi-Fi Direct is suitable for IoT networks, in which a connection between any two devices is created without going through a wireless router or a repeater [8].

For short-distance direct communications, Wi-Fi Direct is comparable with other technologies such as ZigBee and particularly Bluetooth that is widely used for communication between mobile devices. When a Wi-Fi chip that supports IEEE 802.11n is utilized [9], it transmits data at transfer rates as high as 250 Mbps that is 10 times higher than 24 Mbps, the maximum transfer rate of the currently used Bluetooth 4.0. The transfer distance is 100 m, which is ten times longer than that of Bluetooth [10].

Devices connected to the Wi-Fi Direct are classified into the group of owner (GO) and the client. The GO functions as an access point (AP) in an existing Wi-Fi network, while the clients are connected through the GO. Clients can communicate only via the GO, either one-to-one or one to *N* [11]. The GO consumes more energy than the clients, as it has to perform certain functions such as beaconing and forwarding [12]. While research on the method to save the energy in terminals connected to an AP in an existing infrastructure Wi-Fi network has been conducted, there has been little research on the AP power-saving because a constant power supply is assumed for the APs. As the GO plays the role of an AP in the Wi-Fi Direct, it should be portable and is therefore limited by the battery capacity. Thus, the usage of the opportunistic method and the NoA method for power-saving in the GO has been suggested [13].

**Figure 1:**
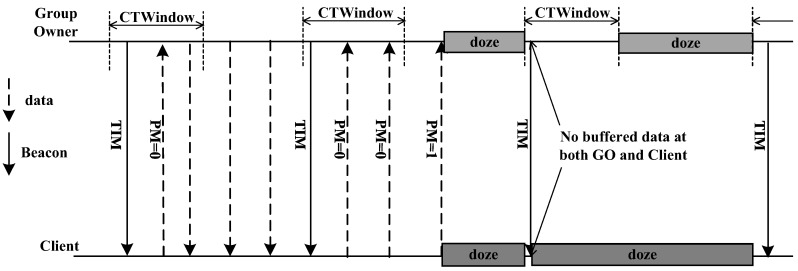
Opportunistic power-saving in Wi-Fi Direct.

Figure 1 shows the opportunistic power-saving method. As the GO sends beacon TIMs (Traffic Indication Messages) regularly to the clients, they are awakened in the section of CT Window (Client Traffic Window) for data transfer. If data transmission is completed before the section of the CT Window ends, the GO and the clients go into the sleep mode upon the end of CT Window. If the GO continues to have more data to send after the end of CT Window, it continues transferring and the clients do not enter the sleep mode until the data transmission is completed. When a client has data to transfer to the GO, the power management bit in the data is set to 0 (PM = 0). At the last data transmission, the power management bit in the data is set to 1 (PM = 1) for notifying that data transmission is completed. If the GO receives a data with PM = 1 during the section of the CT Window, it enters the sleep mode after the Window. If it does not receive a data with PM = 1 even after the CT Window ends, the GO remains awake until it receives the data with PM = 1. Thereafter, it stays in the sleep mode until the next beacon interval [14].

**Figure 2:**
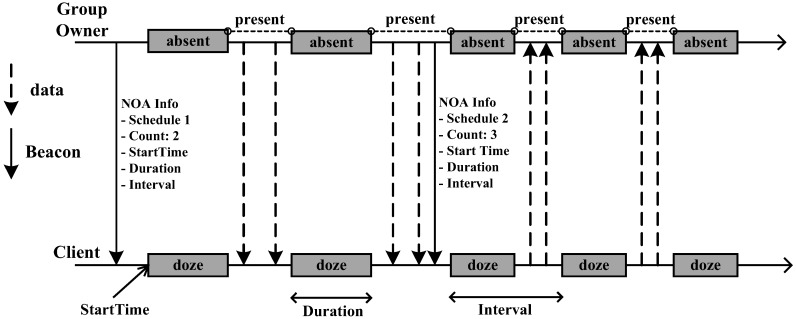
Notice of absence power-saving in Wi-Fi Direct.

Figure 2 shows the NoA method. While the opportunistic method transmits and receives data mainly in the CT Window after the GO sends a beacon, the NoA method *dynamically* determines the number of active/absence periods and its duration in a beacon interval. In the NoA method, the GO sends to the clients the scheduling information of a beacon interval such as the start time of the first absence period, the duration of each absence period, time interval between the absence periods, the number of absence periods in a beacon interval, etc. as part of the beacon message. Clients connect to the GO, then avoid sending data in these absence periods, and the GO enters the sleep mode during the absence periods to save energy [13] .

Various research related to the Wi-Fi Direct power-saving methods has been conducted. In [14], the authors have proposed a new power management system that changes the power management methods depending on the traffic characteristic and have verified through experiments that it is efficient to apply the NoA method for the traffics with periodic characteristic, and to apply the opportunistic method for the traffic with bursty characteristics. In [15], the TAPS (Traffic Awake Parameter tuning Scheme) method that adjusts the parameters dynamically depending upon the traffic load has been proposed and the algorithm to express the GO parameters (CT Window, awake interval, etc.) in traffic flow functions has been developed. Experiment results show that the TAPS method equilibrated the trade-off relationship between the energy and throughput. The author of [12] has suggested two traffic-based dynamic management methods: the adaptive single present period (ASPP) method and the adaptive multiple present period (AMPP) method. These two methods adopt an algorithm that schedules the awake intervals of the GO at each beacon interval based on the traffic activity (utilization) for the purpose of energy saving. Reference [16] proposes an algorithm that takes into account both the energy and the delay for an application with periodic streaming traffics. This algorithm supports two functions for utilization of the existing opportunistic method. The first function is for recognizing the patterns of data sets that are transferred in Wi-Fi Direct frame and the second function is for awakening a device instantly during a sleep interval and receiving periodic data by adjusting the duty cycle dynamically. This function defines the temporary CT window (TCTW) and is applied to the opportunistic method.

In this paper, we propose a method to enhance the energy efficiency of the Wi-Fi Direct power-saving considering the characteristics of variable bit rate (VBR) video traffic. The suggested method determines the number of and the length of awake intervals in a beacon interval reflecting the probability density function (pdf) of the size of video frames so as to reduce unnecessary energy consumption and transmission delay. In addition, the proposed method prioritizes the frame transmission per frame class by considering the inter-dependence between the video frames with the purpose of increasing the success probability of a high priority video frame transmission and thus enhancing the transmission efficiency.

## 2. Video Traffic Model

**Figure 3:**
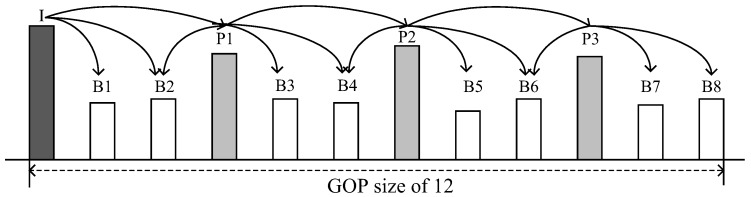
Hierarchical dependency among video frames.

The VBR video traffic consists of three types of video frames: Intra (I), Predictive (P), and Bi-directional (B). As shown in Figure 3, the frames are arranged in pre-determined patterns from an *I*-frame to the next, called a group of picture (GoP) structure. The GoP structure is expressed mainly as MmNn, where n indicates the total number of frames in the GoP and m indicates the *I*-P or *P*-*P*-frame interval [17]. All the frames with the exception of the *I*-frame are encoded based on the dependency among the adjacent frames, while the *I*-frames are encoded as they are without referring to other images. *P*-frames are encoded with reference to the previous *I*-frame or *P*-frame, while *B*-frames refer to the previous and following *I*-frame and (or) *P*-frame. Thus, when an *I*-frame is lost, all frames in the GoP are lost. When a *P*-frame is lost, all of the following *P*- and *B*-frames are lost. In contrast, when a *B*-frame is lost, it does not affect the other frames [18]. To sum up, the listing of the frames in descending order according to their significance is as follows: *I*-frame, *P*-frame, and *B*-frame.

The I-frame gamma autoregressive (I-GAR) model proposed for the asynchronous transfer mode (ATM) network moving picture experts group (MPEG) streams shows some characteristics on the distribution of the video frame size [19]. Since a GoP has only one *I*-frame and *I*-frame is encoded independently regardless of the other frames, *I*-frame does not include a motion compensation functionality that is used to reduce the frame size, so *I*-frame is usually larger than the *B*-frame and *P*-frame in size. Thus, in the *I*-GAR model, the distribution of *I*-frame size is selected as the basis, and it is shown that the pdf of *I*-frame size follows the gamma distribution and the pdfs of the *B*-frame and *P*-frame sizes are expressed also as gamma distributions with the scalar products to the rate parameter of the gamma distribution of *I*-frame size [19].

Let the random variables denoting the size of *I*-frame, *P*-frame and *B*-frame be $Z_{I}$, $Z_{P}$, and $Z_{B}$, respectively. From [19], the pdfs of the each frame size are given by

(1) $$\begin{array}{c} f_{Z_{I}}\left(z\right)=\frac{\lambda^{k}z^{k-1}e^{-\lambda z}}{\Gamma (k)},\;\;\;f_{Z_{P}}\left(z\right)=\frac{{\left(\lambda /m_{P}\right)}^{k}z^{k-1}e^{-(\lambda /m_{P})z}}{\Gamma (k)},\;\;\;f_{Z_{B}}\left(z\right)=\frac{{\left(\lambda /m_{B}\right)}^{k}z^{k-1}e^{-(\lambda /m_{B})z}}{\Gamma (k)}, \end{array}$$

where $m_{P}=\frac{P}{I}=\frac{P1+P2+P3}{I}$ and $m_{B}=\frac{B}{I}=\frac{B1+B2+\ldots +B8}{I}$. Here, the expectations of $Z_{I}$, $Z_{P}$, and $Z_{B}$ are calculated by

(2) $$\begin{array}{c} E\left[Z_{I}\right]=\frac{k}{\lambda },\;\;\;E\left[Z_{P}\right]=\frac{m_{P}k}{\lambda },\;\;\;E\left[Z_{B}\right]=\frac{m_{B}k}{\lambda }. \end{array}$$

The variances of $Z_{I}$, $Z_{P}$, and $Z_{B}$ are calculated by

(3) $$\begin{array}{c} \sigma_{Z_{I}}^{2}=\frac{k}{\lambda^{2}},\;\;\;\sigma_{Z_{P}}^{2}=\frac{m_{P}k}{\lambda^{2}},\;\;\;\sigma_{Z_{B}}^{2}=\frac{m_{B}k}{\lambda^{2}}. \end{array}$$

## 3. Proposed Power-Saving Method for Video Traffic

This chapter explains the proposed power-saving method in the Wi-Fi Direct for the VBR video traffic model. The key idea of the power-saving method proposed in this study is that the scheduling of a beacon interval (the number of and the length of the awake intervals in a beacon interval) is determined dynamically, considering the distribution of the video frame size and the priority of the video frame.

### 3.1. Beacon Interval Scheduling in the Proposed Method

In this subsection, we describe the detailed method to determine the number of awake intervals in a beacon interval and the length of each awake interval. The number of awake intervals in a beacon interval are set so as to satisfy the one-to-one mapping relation between each awake interval and each video frame; i.e., a single awake interval is allotted to one video frame. The length of an awake interval is decided per frame class. Thus, there exists awake intervals for *I*-frames, *P*-frames, and *B*-frames, respectively. Hereafter, we call the awake intervals assigned to *I*-frames, *P*-frames, and *B*-frames as the *I*-frame interval, *P*-frame interval, and *B*-frame interval, respectively. The detailed algorithm to determine the length of the *x*-frame (Here, *x*-frame may be *I*-, *P*-, or *B*-frame) interval is presented as follows.

At first, the GO sets the target probability, $p_{x}$, for *x*-frame. The target probability of the *x*-frame indicates the probability that the *x*-frame can be transmitted wholly in the *x*-frame interval, so there is no segmentation or frame drop due to the short awake interval compared to the frame size. For instance, if the target probability of the *I*-frame, $p_{I}$, is set to 0.95, then it means that the probability of a whole *I*-frame being transferred without any segmentation or frame drop in the *I*-frame interval is 95%, and some *I*-frames cannot be wholly sent in the *I*-frame interval owing to its large size, with a probability of 5%.

From the above definition, once $p_{x}$ is determined, we can get the following expression for a certain positive real number per *x*-frame, $S_{x}$, by

(4) $$p_{x}=\int_{0}^{S_{x}}f_{Z_{x}}\left(z\right)dz.$$

**Figure 4:**
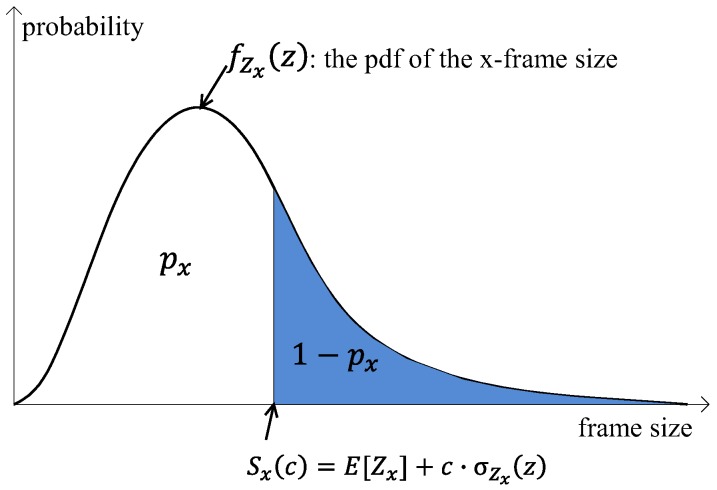
Schematic illustration of $p_{x}$ and $S_{x}\left(c\right)$.

Here, $S_{x}$ indicates the largest size of the *x*-frame being able to be wholly transferred in the *x*-frame interval. $S_{x}$ is a variable decided by the network operator, and, in this study, it is determined with reference to the average and variance of the frame size distribution of each frame class, which is given by

(5) $$S_{x}\left(c\right)=E\left[Z_{x}\right]+c\cdot \sigma_{Z_{x}},$$

where *c* is the scale factor that controls the value of $S_{x}$. The schematic illustration of $p_{x}$ and $S_{x}\left(c\right)$ is given in Figure 4.

Finally, the length of the awake interval for *x*-frame as a function of the scale factor *c* is calculated by

(6) $$T_{x}\left(c\right)=\frac{S_{x}}{v}=\frac{E\left[Z_{x}\right]+c\cdot \sigma_{Z_{x}}}{v},$$

where *v* is the data rate of the given channel.

### 3.2. Frame Transmission Strategy Based on the Frame Priority

**Figure 5:**
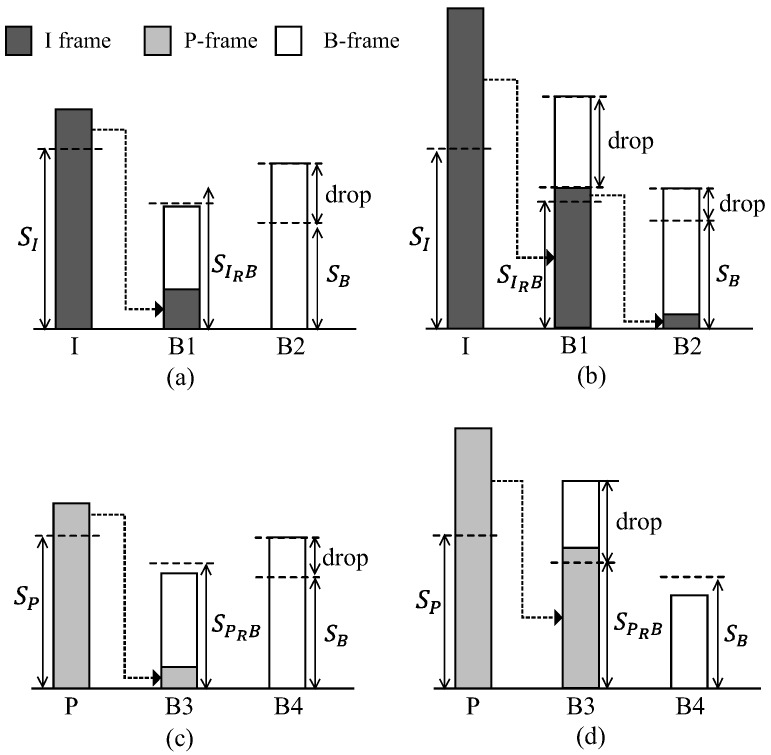
Transmission scheme according to the frame priority: (**a**) remaining *I*-frame is transmitted in the following B1-frame; (**b**) remaining *I*-frame in the B1 frame interval is again concatenated and transmitted in the B2-frame; (**c**) remaining *P*-frame is transmitted in the following B3-frame; (**d**) remaining *P*-frame in the B3 frame interval is dropped.

From the definition of $S_{x}\left(c\right)$, *x*-frames with a size larger than $S_{x}\left(c\right)$ cannot be wholly transmitted during the *x*-frame interval with the probability of $1-p_{x}$. In this case, there exists a remaining fraction of the frame and the method of dealing with the remaining fraction of the frame is different depending upon the priority of each frame. First, *I*-frame has the highest priority, and thus the remaining fraction of the *I*-frame is concatenated with the immediately following B1-frame and transmitted along with the B1-frame in the B1-frame interval, in order to enhance the transmission success probability of the *I*-frame, as seen in Figure 5a. If B1-frame interval is unable to accommodate the remaining fraction of the *I*-frames, the remaining fraction of the *I*-frame in B1-frame interval is again concatenated with the B2-frame and is transmitted in the B2-frame interval, while the whole B1-frame is dropped, as seen in Figure 5b.

As shown in Figure 3, *P*-frame has the second highest priority. Thus, similar to the case of the *I*-frame, the remaining fraction of the *P*-frame not being transferred in its *P*-frame interval is transmitted in the following *B*-frame interval (B3-frame interval in Figure 5c). However, unlike to the case shown in Figure 5b, the proposed method does not transfer the remaining fraction of the *P*-frame not being accommodated in the B3-frame interval to the next *B*-frame interval (B4-frame interval in Figure 5d), but insists on it being dropped.

Finally, the oversized *B*-frames is dropped because of its low priority.

## 4. Algorithm Analysis

### 4.1. Analysis on the Length of the Awake Interval

The remaining fraction of the *I*-frame not being transmitted in the *I*-frame interval is concatenated with the following B1-frame and transferred in the B1-frame interval. Thus, the size of the frame to be transferred during the B1-frame interval becomes the sum of the sizes of the possible remaining fraction of *I*-frame and B1-frame. Thus, to determine the length of the B1-frame interval, it is insufficient to use the B1-frame size distribution in Expression (1) only and a new distribution function is necessary considering the size of the concatenated frame. We denote the remaining fraction of the *I*-frame not being transmitted in the *I*-frame interval as $I_{R}$-frame (*I*-frame residual) and the sum of the *I*-frame residual and B1-frame as the $I_{R}B$-frame. The $P_{R}$-frame (*P*-frame residual) and the $P_{R}B$-frame are defined in the same manner.

Let $Z_{I_{R}}$ be the random variable denoting the size of $I_{R}$-frame. Then, we have

(7) $$Z_{I_{R}}=\left\{\begin{array}{cc} Z_{I}-T_{I}, & \mathrm{if}\;Z_{I}\geq T_{I}\\ 0, & \mathrm{otherwise}. \end{array}\right..$$

Therefore, $Z_{I_{R}}$ becomes the mixed random variable that shares properties of continuous random variable and discrete random variable. Its cumulative density function (cdf) is calculated as follows. When z<0, we trivially have

(8) $$\begin{array}{c} F_{Z_{I_{R}}}\left(z\right)=0. \end{array}$$

When z=0, we have

(9) $$F_{Z_{I_{R}}}\left(0\right)=P\left[Z_{I_{R}}=0\right]=P\left[Z_{I}<S_{I}\left(k\right)\right]=p_{I}.$$

When z>0, we have

(10) $$\begin{array}{cc} F_{Z_{I_{R}}}\left(z\right) & =P[Z_{I_{R}}\leq z]\\ & =P\left[Z_{I_{R}}\leq z,Z_{I}\geq S_{I}\left(c\right)\right]+P\left[Z_{I_{R}}\leq z,Z_{I}<S_{I}\left(c\right)\right]\\ & =P\left[S_{I}\left(c\right)\leq Z_{I}\leq z+S_{I}\left(c\right)\right]+P\left[Z_{I}<S_{I}\left(c\right)\right]\\ & =F_{Z_{I}}\left(z+S_{I}\left(c\right)\right). \end{array}$$

From Equations (6)–(8), the pdf of $Z_{I_{R}}$ is obtained by

(11) $$f_{Z_{I_{R}}}\left(z\right)=f_{Z_{I}}\left(z+S_{I}\left(c\right)\right)+p_{I}\cdot \delta \left(z\right),$$

where δ(·) is a Dirac−Delta function. Let $Z_{I_{R}B}$ be the random variable denoting the sizes of $I_{R}B$ frame. Because $Z_{I_{R}B}=Z_{I_{R}}+Z_{B}$, the pdf of $Z_{I_{R}B}$, $f_{Z_{I_{R}B}}$, becomes the convolution of $f_{Z_{I_{R}}}\left(z\right)$ and $f_{Z_{B}}\left(z\right)$ and is calculated by

(12) fZIRB(z)=FZI(SI(c))(λSB)kzk−1e−(λmB)zΓ(k)+(1mB)kλ2ke−λ(z+SI(c))Γ(k)2∑i=0k−1(−1)k−1−ik−1i(z+SI(c))i·[−∑j=02k−2−ieZIRB(λ−λSB)[−(λ−λSB)]j+1(2k−2−i)!(2k−2−i−j)!+(2k−2−i)![−(λ−λSB)]2k−1−i].

The detailed calculation of $f_{Z_{I_{R}B}}\left(z\right)$ is given in Appendix A.1. Since the sizes of $I_{R}$-frame and *B*-frame are independent each other, $E[Z_{I_{R}B}]$, the average of $I_{R}B$-frame size distribution can be expressed by the sum of average sizes of $I_{R}$-frame and *B*-frame, so $E\left[Z_{I_{R}B}\right]=E\left[Z_{I_{R}}\right]+E\left[Z_{B}\right]$. Now, the average of $I_{R}$-frame size is calculated by

(13) E[ZIR]=λke−λSI(c)Γ(k)∑j=0k−1k−1jSI(c)k−1−j(j+1)!λj+2,

where the detailed calculation is given in Appendix A.2. Thus, the average distribution of $I_{R}B$-frame is given by

(14) E[ZIRB]=E[ZIR]+E[ZB]=λke−λSI(c)Γ(k)∑j=0k−1k−1jSI(c)k−1−j(j+1)!λj+2+kmBλ.

From Appendix A.3, $E[{Z^{2}}_{I_{R}}]$ is calculated by

(15) E[Z2IR]=λke−λSI(c)Γ(k)∑j=0k−1k−1jSI(c)k−1−j(j+2)!λj+3.

Therefore, the variance of $I_{R}$-frame, ${\sigma^{2}}_{I_{R}}$, is calculated by

(16) σ2IR=E[Z2IR]−(E[ZIR])2=λke−λSI(c)Γ(k)∑j=0k−1k−1jSI(c)k−1−j(j+2)!λj+3−(λke−λSI(c)Γ(k)∑j=0k−1k−1jSI(c)k−1−j(j+1)!λj+2)2.

The variance of $Z_{I_{R}B}$, is calculated by

(17) σ2ZIRB=σ2IR+σ2ZB=λke−λSI(c)Γ(k)∑j=0k−1k−1jSI(c)k−1−j(j+2)!λj+3−(λke−λSI(c)Γ(k)∑j=0k−1k−1jSI(c)k−1−j(j+1)!λj+2)2+kmBλ2.

Combining Equations (6), (14), and (17) determines the length of the $I_{R}B$-frame interval. Similarly to Equation (12), the distribution of $P_{R}B$-frame size is obtained by convolution of $f_{P_{R}}\left(z\right)$ and $f_{B}\left(z\right)$ and is given by

(18) fZPRB(z)=FZP(SP(c))(λmP)kzk−1e−λmPzΓ(k)+λ2k(1mBmP)ke−λmP(z+SP(c))Γ(k)2∑i=0k−1(−1)k−1−jk−1i(z+SP(c))i·−∑j=02k−2−iez(λmP−λmB)[−(λmP−λmB)(j+1)](2k−2−i)!(2k−2−i−j)!z2k−2−i−j+(2k−2−i)![−(λmP−λmB)](2k−1−i).

The average $P_{R}$-frame size is calculated by

(19) E[ZPR]=(λmP)ke−λmPSP(c)Γ(k)∑j=0k−1k−1jSP(c)k−1−j(j+1)!(λmP)j+2.

The average $P_{R}B$-frame size is given by

(20) E[ZPRB]=E[ZPR]+E[ZB]=(λmP)ke−λmPSP(c)Γ(k)∑j=0k−1k−1jSP(c)k−1−j(j+1)!(λmP)j+2+kmBλ.

$E[{Z^{2}}_{P_{R}}]$, ${\sigma^{2}}_{P_{R}}$ and ${\sigma^{2}}_{Z_{P_{R}B}}$ are calculated as follows:

(21) E[Z2PR]=(λmP)ke−λmPSP(c)Γ(k)∑j=0k−1k−1jSP(c)k−1−j(j+2)!(λmP)j+3,

(22) σ2ZPR=E[Z2PR]−(E[ZPR])2=(λmP)ke−λmPSP(c)Γ(k)∑j=0k−1k−1jSP(c)k−1−j(j+2)!(λmP)j+3−((λmP)ke−λmPSP(c)Γ(k)∑j=0k−1k−1jSP(c)k−1−j(j+1)!(λmP)j+2)2,

(23) σ2ZPRB=σ2ZPR+σ2ZB=(λmP)ke−λmPSP(c)Γ(k)∑j=0k−1k−1jSP(c)k−1−j(j+2)!(λmP)j+3−((λmP)ke−λmPSP(c)Γ(k)∑j=0k−1k−1jSP(c)k−1−j(j+1)!(λmP)j+2)2+kmBλ2.

Then, we can obtain the length of the awake interval for $P_{R}B$-frame from Equations (6), (20), and (23). It is noted that the lengths of the awake interval for ordinary *I*-frame, *P*-frame, and *B*-frame are easily obtained from Equations (2), (6) and (3).

### 4.2. Delay and Energy Consumption

We define the average delay per frame as the ratio of total sum of delay time of frames to the number of total frames in a given time period. To obtain the average delay, we calculate the probability of each frame not being transmitted during its awake interval, as follows:

(24) $$\begin{array}{cc} {\bar{P}}_{I} & =P(Z_{I}>E\left[Z_{I}\right]+c\cdot \sigma_{Z_{I}})\\ & =1-\frac{\lambda^{k}}{\Gamma (k)}\left[\sum_{i=0}^{k-1}\frac{e^{-\lambda S_{I}\left(c\right)}}{\lambda^{i+1}}\frac{(k-1)!}{(k-1-i)!}S_{I}{\left(c\right)}^{k-1-i}-\frac{(k-1)!}{\lambda^{k}}\right], \end{array}$$

(25) $$\begin{array}{cc} {\bar{P}}_{P} & =P(Z_{P}>E\left[Z_{P}\right]+c\cdot \sigma_{Z_{P}})\\ & =1-\frac{{\left(\frac{\lambda }{m_{P}}\right)}^{k}}{\Gamma (k)}\left[\sum_{i=0}^{k-1}\frac{e^{-\frac{\lambda }{m_{P}}S_{P}\left(c\right)}}{{\left(\frac{\lambda }{m_{P}}\right)}^{i+1}}\frac{(k-1)!}{(k-1-i)!}{\left(S_{P}\left(c\right)\right)}^{k-1-i}-\frac{(k-1)!}{{\left(\frac{\lambda }{m_{P}}\right)}^{k}}\right], \end{array}$$

(26) $$\begin{array}{cc} {\bar{P}}_{B} & =P(Z_{B}>E\left[Z_{B}\right]+c\cdot \sigma_{Z_{B}})\\ & =1-\frac{{\left(\frac{\lambda }{m_{B}}\right)}^{k}}{\Gamma (k)}\left[\sum_{i=0}^{k-1}\frac{e^{-\frac{\lambda }{m_{B}}S_{B}\left(c\right)}}{{\left(\frac{\lambda }{m_{B}}\right)}^{i+1}}\frac{(k-1)!}{(k-1-i)!}{\left(S_{B}\left(c\right)\right)}^{k-1-i}-\frac{(k-1)!}{{\left(\frac{\lambda }{m_{B}}\right)}^{k}}\right]. \end{array}$$

Let *f* be the inter-frame interval of the GoP structure. Then, the delay time of the oversized *I*-frame and *P*-frame become $f-T_{I}\left(c\right)$ and $f-T_{I}\left(c\right)$, respectively. Then, the average delay per frame is given by

(27) $$D_{ave}=\frac{{\bar{P}}_{I}\sum_{i=1}^{N_{I}}\left(f-T_{I}\left(c\right)\right)+{\bar{P}}_{P}\sum_{i=1}^{N_{P}}\left(f-T_{p}\left(c\right)\right)}{N},$$

where *N*, $N_{I}$, and $N_{P}$ are the numbers of total frames, *I*-frame, *P*-frame, in a given GoP structure, respectively.

The average energy consumption per frame is defined as the ratio of the total energy consumption used to transfer all frames in a GoP to the number of frames in a GoP. Since the number of $I_{R}B$($P_{R}B$)-frame is the same as the number of *I*(*P*)-frame, the average energy consumption per frame using the proposed method is given by:

(28) $$\begin{array}{cc} E_{ave} & =\frac{P_{sleep}}{N}\left(\sum_{i=1}^{N_{I}}\left(f-T_{I}\right)+\sum_{i=1}^{N_{P}}\left(f-T_{P}\right)+\sum_{i=1}^{N_{I}}\left(f-T_{I_{R}B}\right)+\sum_{i=1}^{N_{P}}\left(f-T_{P_{R}B}\right)+\sum_{i=1}^{N_{B}-N_{I}-N_{P}}\left(f-T_{B}\right)\right)\\ & \;\;\;\;+\frac{P_{awake}}{N}\left(\sum_{i=1}^{N_{I}}T_{I}+\sum_{i=1}^{N_{P}}T_{P}+\sum_{i=1}^{N_{I}}T_{I_{R}B}+\sum_{i=1}^{N_{P}}T_{P_{R}B}+\sum_{i=1}^{N_{B}-N_{I}-N_{P}}T_{B}\right)+E_{switch}, \end{array}$$

where $P_{sleep}$ and $P_{awake}$ mean the energy consumption of a mobile device per unit-time when they are in sleep mode and awake mode, respectively, and $N_{B}$ is the number of *B*-frames in a GoP. In addition, $E_{switch}$ means the energy consumption for switching mode from sleep to awake in the network interface card (NIC). Switching mode from sleep to awake occur *N* times in a GoP; therefore, the additional power consumption to transfer a frame is calculated as $E_{switch}$, respectively. Since both of the lengths of the awake interval and the sleep interval in the NoA method are fixed, the average energy consumption per frame using the NoA scheme is expressed by

(29) $$E_{ave}=P_{awake}T_{NoA}+P_{sleep}\left(f-T_{NoA}\right)+E_{switch},$$

where $T_{NoA}$ means the length of the awake interval in the NoA method.

## 5. Results

To evaluate the performance of the proposed method, we execute the Monte Carlo simulation runs with 20,000 GoP frames. The implementation of a testbed by using real devices reveals additional constraints issues in relation to power consumption due to the limitations of devices [20] and additional power consumption such as standby power of devices [21]. In this paper, the validity of the proposed method is verified via numerical method and simulation in order to exclude the impact of the additional constraints in relation to power consumption and focus on the performance evaluation of the proposed method. The simulation setup is as follows: as VBR traffic, a video codec with 25 fps and the GoP pattern of $I{\left(BBP\right)}^{3}BB$ is assumed. Therefore, we have $N_{I}=1$, $N_{P}=3$, and $N_{B}$ = 8. The frame interval in a GoP structure, *f*, is set to 40 ms, and the beacon interval is set to 120 ms so that each beacon interval could transfer consecutive three video frames. From [19], k=22.39826 and γ=44.97535 are used for the gamma distribution of *I*-frame size. The scale factors for the distribution of the *B*- and *P*-frame sizes are set as $m_{P}=0.26262$ and $m_{B}=0.13273$, respectively. The channel speed is set to 6 Mbps from [22]. The values of $P_{sleep}$ and $P_{awake}$ are obtained from [12], and $E_{switch}$ is obtained from [20]. Simulation parameters used in our study is summarized in Table 1.

For the performance evaluation, we measure the average delay per frame and the average energy consumption per frame using both the proposed method and the NoA method. Here, we compare the performance of the proposed method with that of the NoA method other than the opportunistic method because the video traffic considered in this study is predictable in the sense that the traffic is generated periodically, and is therefore more suitable for the NoA method than the opportunistic method.

**Figure 6:**
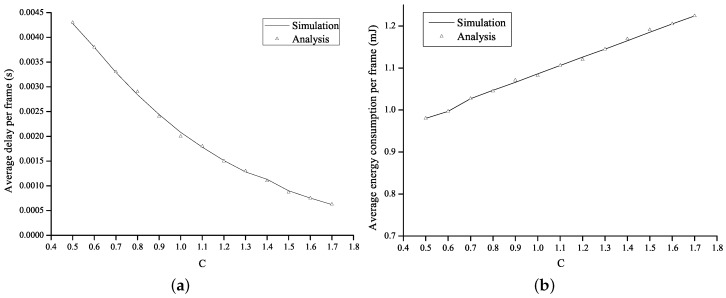
Performance of the proposed method. (**a**) average delay per frame; and (**b**) average energy consumption per frame.

Figure 6 shows the average energy consumption per frame and the average delay per frame as a function of the value of *c* in the proposed method. The operational parameter *c* is set to range from 0.5 to 1.7. Figure 6a shows that, as *c* increases, the awake interval is extended so that the number of the frame transmission failure decreases. As a result, the average delay is shortened. However, as the awake interval is extended in proportion to the value of *c*, more energy was consumed, as shown in Figure 6b.

**Figure 7:**
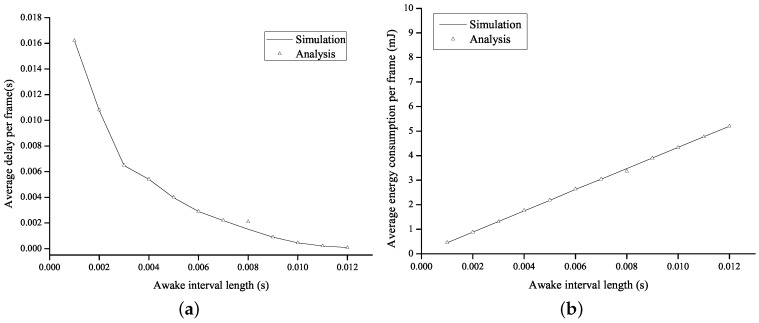
Performance of the NoA method. (**a**) average delay per frame; and (**b**) average energy consumption per frame.

Figure 7 shows the average energy consumption per frame and the average delay per frame as a function of the length of the awake interval in the NoA method. In the proposed method, the shortest and the longest awake intervals correspond to the *B*-frame interval with c=0.5 and the *I*-frame interval with c=1.7, respectively. To compare the performance of the NoA method with that of the proposed method under the same environment, the length of the awake interval in the NoA method is set to a range of 1–12 ms, which includes the shortest and the longest awake intervals in the proposed method. We can verify the trade-off relation between the energy consumption of a node and the transmission delay from Figure 7, so that the average energy consumption increases as the length of the awake interval increases and the average delay decreases accordingly.

**Figure 8:**
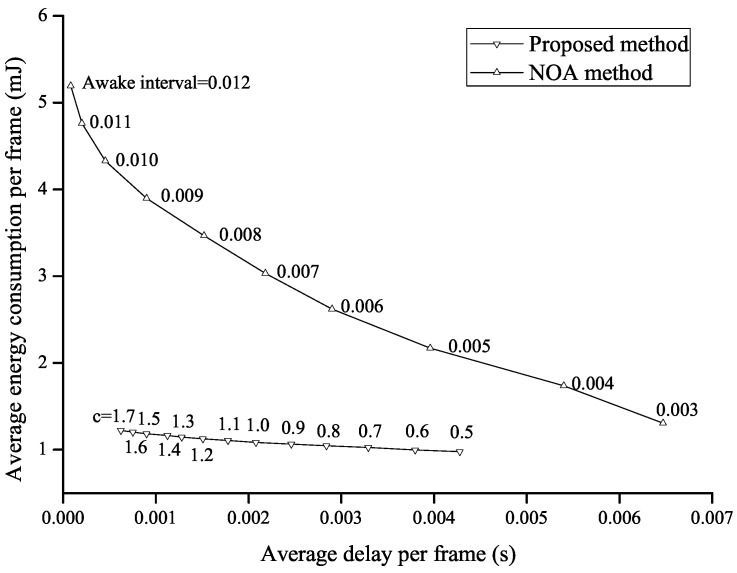
Performance comparison between the proposed and NoA methods.

In Figure 8, the performance of the proposed method is directly compared to that of the NoA method in terms of the average delay per frame and the average energy consumption per frame. Every point on each curve can be obtained by varying the value of *c* in the proposed method or by varying the length of the awake interval in the NoA method. The energy-delay trade-off relation is shown in both of the methods, and the energy consumption decreases as the average delay increases. It is noticed that the inner curve shows better tradeoff performances, i.e., both the energy consumption and the delay are smaller than those of the outer curve. The outer curve is given by the NoA method, and the inner curve is given by the proposed method. This implies that the proposed method enhances the overall performance and shows better performance than the NoA method.

## 6. Conclusions

In this paper, we propose an algorithm for scheduling a beacon interval based on the video frame size distribution in order to enhance energy efficiency. The proposed method schedules a beacon interval so that one awake interval is mapped to one video frame, and determines the length of the awake interval per frame class considering the distribution functions of the sizes of *I*-frame, *P*-frame, and *B*-frame. In addition, considering the fact that video frames are prioritized due to mutual dependency, it is set that a frame having high priority is transmitted with higher probability than a frame having lower priority by making the remaining fraction of the high priority frame be concatenated and transmitted with the following next frame. Simulation runs are conducted to compare the performance of the proposed method with that of the NoA method, and it is shown that the proposed method shows a better performance than the NoA method in terms of the average energy consumption and the average delay. On the other hand, the proposed algorithm in this work assumed that there is a single client associated with the GO. However, there may be multipl clients that share the same traffic characteristics, and thus it would be our important future work to extend the proposed algorithm for multiple clients. Furthermore, we have a plan to develop the appropriate power-saving algorithm for various traffic other than video traffic, which will be another important future work. In addition, we will evaluate the performance of proposed method in a real testbed in order to verify the validity of the proposed method and possible constraints in relation to power consumption.

multiple

## Figures and Tables

**Table 1:** Simulation parameters.

Parameter	Value	Parameter	Value
frame interval (*f*)	40 ms	channel rate (*v*)	6 Mbps
*N*	12	$N_{I}$	1
$N_{P}$	3	$N_{B}$	8
Beacon Interval	120 ms	count	3
$P_{awake}$	432 mW	$P_{sleep}$	0.3 mW
$P_{switch}$	0.6 uJ		

## Appendix A

In this appendix, we provide the detailed derivation of the pdf of random variable $Z_{I_{R}B}$, its mean and second moment.

### Appendix A.1

The convolution of $Z_{R_{R}}$ and $Z_{B}$ is calculated by

(A1) fZIRB(z)=(fZIR*fZB)(z)=∫0zfZIR(z−τ)fZB(τ)dτ=∫0zλk((z−τ+SI(c)))k−1e−λzΓ(k)+pIδ(z−τ)(λmB)kzk−1e−λmBzΓ(k)dτ=∫0zλk((z−τ+SI(c)))k−1e−λzΓ(k)(λmB)kzk−1e−λmBzΓ(k)+pIδ(z−τ)(λmB)kzk−1e−λmBzΓ(k)dτ=∫0zpIδ(z−τ)(λmB)kzk−1e−λmBzΓ(k)dτ+∫0zλk((z−τ+SI(c)))k−1e−λzΓ(k)(λmB)kzk−1e−λmBzΓ(k)dτ=pI(λSB)kzk−1e−(λSB)zΓ(k)+(1mB)kλ2ke−λ(z+TI)Γ(k)2∫0zτk−1(z−τ+SI(c))k−1eτ(λ−λmB)dτ=FZI(SI(c))(λmB)kzk−1e−λmBzΓ(k)+(1mB)kλ2ke−λ(z+SI(c))Γ(k)2∫0zτk−1(z−τ+SI(c))k−1eτ(λ−λmB)dτ=FZI(SI(c))(λmB)kzk−1e−λmBzΓ(k)+(1mB)kλ2ke−λ(z+SI(c))Γ(k)2∑i=0k−1(−1)k−1−ik−1i(z+SI(c))i∫0zτ2k−2−ieτ(λ−λmB)dτ=FZI(SI(c))(λmB)kzk−1e−λmBzΓ(k)+(1mB)kλ2ke−λ(z+SI(c))Γ(k)2∑i=0k−1(−1)k−1−ik−1i(z+SI(c))i·−∑j=02k−2−ie(λ−λmB)z[−(λ−λmB)]j+1(2k−2−i)!(2k−2−i−j)!z(2k−2−i−j)+(2k−2−i)![−(λ−λmB)](2k−1−i).

### Appendix A.2

The expectation value of $Z_{I_{R}}$ is calculated by

(A2) E[ZIR]=E[ZIR∣ZI≥SI(c)]+E[ZIR∣ZI<SI(c)]P[ZI<SI(c)]=(1−pI)·∫0∞zfZIR∣ZI≥SI(c)(z∣ZI−SI(c))dz+pI·0=(1−pI)·∫0∞zfZIR(z)1−pIdz=∫0∞zfZIR(z)dz=∫0∞zfZI(z+SI(c))dz=∫0∞zλk(z+SI(c))k−1e−λ(z+SI(c))Γ(k)dz=λke−λSI(c)Γ(k)∫0∞z(z+SI(c))k−1e−λzdz=λke−λSI(c)Γ(k)∫0∞z∑j=0k−1k−1jzjSI(c)k−1−je−λzdz=λke−λSI(c)Γ(k)∑j=0k−1k−1jSI(c)k−1−j∫0∞zj+1e−λzdz=λke−λSI(c)Γ(k)∑j=0k−1k−1jSI(c)k−1−j(j+1)!λj+2.

### Appendix A.3

The second moment of $Z_{I_{R}}$ is calculated by

(A3) E[ZIR2]=E[ZIR2∣ZI≥SI(c)]P[ZI≥SI(c)]+E[ZIR2∣ZI<SI(c)]P[ZI<SI(c)]=(1−pI)·∫0∞z2fZIR∣ZI≥SI(c)(z∣ZI−SI(c))dz+pI·0=(1−pI)·∫0∞z2fZIR(z)1−pIdz=∫0∞z2fZIR(z)dz=∫0∞z2fZI(z+SI(c))dz=∫0∞z2λk(z+SI(c))k−1e−λ(z+SI(c))Γ(k)dz=λke−λSI(c)Γ(k)∫0∞z2(z+SI(c))k−1e−λzdz=λke−λSI(c)Γ(k)∫0∞z2∑j=0k−1k−1jzjSI(c)k−1−je−λzdz=λke−λSI(c)Γ(k)∑j=0k−1k−1jSI(c)k−1−j∫0∞zj+2e−λzdz=λke−λSI(c)Γ(k)∑j=0k−1k−1jSI(c)k−1−j(j+2)!λj+3.
